# Evaluating the Single-Shot MultiBox Detector and YOLO Deep Learning Models for the Detection of Tomatoes in a Greenhouse

**DOI:** 10.3390/s21103569

**Published:** 2021-05-20

**Authors:** Sandro Augusto Magalhães, Luís Castro, Germano Moreira, Filipe Neves dos Santos, Mário Cunha, Jorge Dias, António Paulo Moreira

**Affiliations:** 1 INESC TEC-Instituto de Engenharia de Sistemas e Computadores, Tecnologia e Ciência, Campus da FEUP, Rua Dr. Roberto Frias, s/n, 4200-465 Porto, Portugal; luis.r.castro@inesctec.pt (L.C.); mccunha@fc.up.pt (M.C.); amoreira@fe.up.pt (A.P.M.); 2 Faculty of Engineering, University of Porto, Rua Dr. Roberto Frias, s/n, 4200-465 Porto, Portugal; 3 Faculty of Sciences, University of Porto, Rua do Campo Alegre, s/n, 4169-007 Porto, Portugal; up201608269@fc.up.pt; 4 Khalifa University Center for Autonomous Robotic Systems (KUCARS), Khalifa University of Science and Technology (KU), Abu Dhabi 127788, United Arab Emirates; jorge.dias@ku.ac.ae

**Keywords:** vision system, object detection, fruit detection, machine learning, SSD benchmarking, robotics vision

## Abstract

The development of robotic solutions for agriculture requires advanced perception capabilities that can work reliably in any crop stage. For example, to automatise the tomato harvesting process in greenhouses, the visual perception system needs to detect the tomato in any life cycle stage (flower to the ripe tomato). The state-of-the-art for visual tomato detection focuses mainly on ripe tomato, which has a distinctive colour from the background. This paper contributes with an annotated visual dataset of green and reddish tomatoes. This kind of dataset is uncommon and not available for research purposes. This will enable further developments in edge artificial intelligence for in situ and in real-time visual tomato detection required for the development of harvesting robots. Considering this dataset, five deep learning models were selected, trained and benchmarked to detect green and reddish tomatoes grown in greenhouses. Considering our robotic platform specifications, only the Single-Shot MultiBox Detector (SSD) and YOLO architectures were considered. The results proved that the system can detect green and reddish tomatoes, even those occluded by leaves. SSD MobileNet v2 had the best performance when compared against SSD Inception v2, SSD ResNet 50, SSD ResNet 101 and YOLOv4 Tiny, reaching an F1-score of 66.15%, an mAP of 51.46% and an inference time of 16.44 ms with the NVIDIA Turing Architecture platform, an NVIDIA Tesla T4, with 12 GB. YOLOv4 Tiny also had impressive results, mainly concerning inferring times of about 5 ms.

## 1. Introduction

Tomatoes, grown in different crop systems, are the world’s second-most harvested vegetable and the leader among greenhouse vegetables [1]. In the last few decades, greenhouse tomato cultivation in several systems has increased worldwide because it has the advantage of enabling high productivity and stable supply throughout the year.

While the value of greenhouse tomatoes is high on a per-unit basis, the costs are also high, mainly due to the labour costs. Greenhouse’s manual operations account for up to 50% of the total greenhouse production costs, a large part of these costs being absorbed by the manual tomato harvesting, which requires 700 $h\;{\mathrm{yr}}^{-1}\;{\mathrm{ha}}^{-1}$ to 1400 h ${\mathrm{yr}}^{-1}\;ha$^−1^ according to the cropping system [2,3]. Hence, manual harvesting of tomatoes is, actually, a challenge due to the global labour shortage and precarious working conditions [2,4,5]. Moreover, farmers need to secure additional workers during harvest seasons because the manpower requirements are higher than usual during this time [5]. Therefore, reducing the amount of labour in a greenhouse company is an important topic at the moment, and many companies are looking for automated solutions, such as harvesting robots [5].

**Figure 1:**
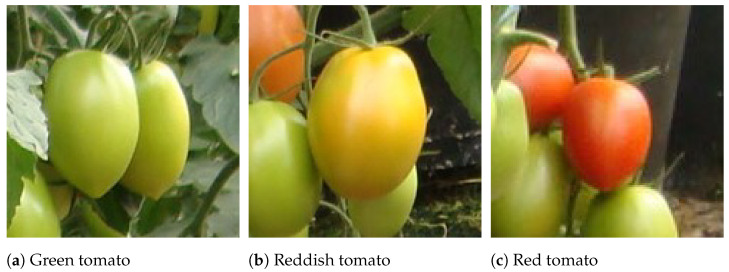
Tomatoes’ ripeness levels: (**a**) physiological or horticultural maturation; (**b**) early phase of ripening; and (**c**) ripened tomato.

The development of harvesting robots to work in greenhouses is always challenging because robots have to work in unstructured environments and perform uncertain tasks. In the case of harvesting tomato, the sensing mechanism has to detect fruit in the presence of various disturbances in an unpredicted heterogeneous environment included different arrangements of plant sizes and shapes, stems, branches, leaves, variable colour, under leaves, sun glare and variable light conditions [6,7]. Even more, as a climacteric fruit, according to the technological objectives, the tomato can be harvested at the physiological maturity phase (green colour), completing the ripening after harvest, or it can be harvested later at different stages of ripening in which it already has a reddish colour [8]. When to harvest will depend on how the tomato will be handled and used. Fresh market fruit for local consumers can be picked red, while fruit that will be transported long distances should be harvested at early maturation with green colour (Figure 1).

Accurately identifying and detecting the mature fruit or fruit bunches comprise a key technique of harvesting robot research, which has recently received considerable scientific scrutiny. The performance of tomato harvesting robots has been greatly improved by the use of Artificial Intelligence (AI) tools mainly in tomato detection on images acquired in varying environmental and growth conditions such as fruit partially hidden by leaf or stem, state of ripeness (coloration) and light conditions.

The current State-of-the-Art (SoA) explores and proposes different strategies to classify and detect tomatoes based on RGB images. However, in a general overview, these strategies are mostly framed in Machine Learning (ML) applications. These strategies are divided essentially based on the application of the most classical ML algorithms, using, for instance, Support Vector Machines (SVMs) [9] or Deep Learning (DL) strategies [10]. DL is based on the training of Artificial Neural Network (ANN) models to recognise features on annotated images (usually referred to as the training set). Then, the trained model is used to detect the trained objects on new images. The state-of-the-art identifies different ANN model structures, such as:

(i) Convolutional Neural Networks (CNNs);
(ii) Single-Shot MultiBox Detectors (SSDs).

In comparison to other state-of-the-art ANN structures, the SSD [11] aims to be faster. Besides, the SSD does not compromise on the detection accuracy [11].

This work purposes the development of a vision system to recognise tomatoes in real scenarios by using a dataset of tomatoes grown in a greenhouse. The system explores DL models to be run in a TensorFlow Processing Unit (TPU) [12], to assure a high-speed tomato detection system. Currently, only the SSD and some YOLO models can run inside the TPU, and we benchmarked five SSD and YOLO pre-trained models in the COCO dataset [13] and the Open Images Dataset (OID) [14]:

(i) SSD MobileNet v2;
(ii) SSD Inception v2;
(iii) SSD ResNet 50;
(iv) SSD ResNet 101;
(v) YOLOv4 Tiny [15].

The paper’s structure is based on five sections, complemented by the current Introduction section. Section 2 reviews of the related work that contributes to the experiment and states the needed background to understand it. Section 3 explains the experiment, i.e., how we gathered the data and pre-processed them and how the experiment was performed. Section 4 presents the results and performs a deep analysis to understand the best deep learning models for tomatoes’ detection. Finally, Section 5 summarises the experiments and the results and indicates the future work to improve these results towards the online detection of tomatoes for harvesting or monitoring purposes.

## 2. State-of-the-Art

### 2.1. Literature Review

This section presents various algorithms, methods and techniques that were proposed and used by different authors regarding fruit detection, more specifically the tomato (Table 1).

In recent years, machine learning, and especially deep learning, techniques in fruit detection has been increasingly used and tested [9,16,17,18,19,20,21,22,23,24,25,26,27,28,29,30,31,32,33]. Unlike conventional methods, machine learning is a more robust and accurate alternative with a better response to problems such as occlusion and green tomato detection. The problem of green tomato detection is rarely studied due to the difficulty of segmentation and differentiating it from the background, as they have similar colours. This can be observed by the comparison made by E Alam Siddiquee et al. [34]. They compared a machine learning method, known as the “cascaded object detector”, with a system that combines more traditional methods of image processing: “colour transformation”, “colour segmentation”, and “circular Hough transformation”, in the detection of ripe tomatoes. The results showed that the accuracy of the machine learning method is 95% better than conventional methods.

For the detection and segmentation purposes of tomatoes in plants, authors usually consider the plants’ canopy as the Region of Interest (RoI) (see Figure 7). In this RoI, besides the fruits, other structures may be seen that make the fruits’ detection difficult—relevant to estimate the fruit location. These other structures may occlude or overlap the tomato/fruit, and this creates some challenges for the algorithms or processes that are responsible for the fruit detection and segmentation. These challenges become greater in the early ripening stages, due to the high colour correlation between leaves and tomatoes. Despite this fact, the most common and relevant research found in the literature considers the harvesting period in the late maturation stage of tomatoes (where the tomato is already red), so the colour is therefore a feature used recurrently to differentiate the objects to be detected [16,17,18,19,20,21,25,35]. Considering the case of fruit detection and segmentation, the authors try to distinguish it from everything external and the background, which at the crop level, can be very complex. Several colour spaces such as Hue, Saturation and Intensity (HSI) [18,19], CIELAB (or L*a*b*) [16,17,25] and RGB [18,19,20,35] are used to extract this feature. Besides, mathematical morphology approaches [36] combined with machine learning techniques have also been used in fruit detection in occlusion and overlap situations [9,16,17,18,19,20,21,22,23,24,25,26,27].

For developing a harvesting robot in greenhouses, Yin et al. [16] segmented ripe tomatoes through K-means clustering using the CIELAB colour space, recording an average task execution time of 10.14 s. Huang et al. [17] used the CIELAB colour space to segment and localise ripe tomatoes in a greenhouse and bi-level partition fuzzy logic entropy to discriminate the fruits from the background. They did not evaluate the performance of the algorithm. Zhao et al. [25] developed a detection algorithm capable of recognising green, or intermediate, and ripe tomatoes. First, the images of component a* and the images of component L* were extracted from the colour space L*a*b* and the luminance of the Quadrature-phase (YIQ) colour space, respectively. Then, wavelet transformation was adopted to merge the images at the pixel level, which combined the information from the two original images. Finally, to differentiate the fruits from the background, an adaptive threshold algorithm was used to obtain the optimal threshold. The evaluation tests proved a detection rate of 93% of tomatoes.

Arefi et al. [18] proposed an algorithm for the recognition of ripe tomatoes through a combination of the RGB, HSI and YIQ colour spaces and the morphological characteristics of the image. The algorithm obtained a total accuracy of 96.36% when tested in a greenhouse with artificial lighting. Feng et al. [35] developed a ripe tomato harvesting robot for a greenhouse, whose identification and location of fruits consisted of the transformation of the RGB colour space images into an HSI colour model to identify and locate the fruits. The robot performed this task in 4 s, and the harvest process had a success rate of 83.9%. Zhang [19], aiming to detect ripe tomatoes, also converted the RGB colour space into an HSI colour space. The ripe tomato region was cut based on the grey distribution of the H component using the threshold method. The Canny operator [37] was used to detect the edges. After a corrosive expansion, the coordinates of the centre of the tomato were marked. The results were not quantified. Benavides et al. [20] designed a computer vision system for the detection of ripe tomatoes in greenhouses. The segmentation of the fruit was mainly done based on the colour and edges of the fruit, using the R component of the RGB images and the Sobel operator [38], respectively. Clustered tomatoes were detected with a precision of 87.5% and beef tomatoes with 80.8%. Malik et al. [21] presented a ripe tomato detection algorithm based on the HSV (Hue, Saturation, Value) colour space and the watershed segmentation method. For removing the background and detecting only ripe tomatoes, the HSV colour space was used, and through morphological operations, it was possible to modify the detected fruits. The watershed segmentation algorithm was implemented to “separate” the clustered fruits. The combination of these two methods led to a precision of 81.6%.

Zhu et al. [22] combined the mathematical morphology with a Fuzzy C-Means (FCM)-based method for the detection of ripe tomatoes in a greenhouse, with no results reported. Based on the mathematical morphology, Xiang et al. [23] tested a ripe cluster tomato recognition algorithm. The algorithm was divided into four fundamental steps: tomato image segmentation, performed based on a normalised colour difference; recognition of the clustered region according to the length of the longest edge of the minimum enclosing rectangle of the tomato region; clustered regions, in a binary image, were processed by an iterative erosion course to separate each tomato in this clustered region, and every seed region in the clustered region acquired by the iterative erosion was restored using a circulatory dilation operation. At a distance of 500 mm, they achieved a detection rate of 87.5%, while from 300 mm to 700 mm, the rate dropped to 58.4%.

Yamamoto et al. [24] used different machine learning techniques to detect and distinguish the different stages of tomato ripeness. The proposed method consisted of three steps: pixel-based segmentation conducted to roughly segment the pixels of the images into classes composed of fruits, leaves, stems, and background; blob-based segmentation to eliminate the wrong classifications generated in the first step; and finally, X-means clustering was applied to detect fruits individually in a fruit cluster. The results indicated a precision of 88%. Zhao et al. [39], to detect ripe tomatoes, extracted the Haar-like features of the grey-scale image, classifying them with the AdaBoost classifier. The false negatives that derived from this classification were eliminated using a colour analysis approach based on the average pixel value (APV). The results showed that the combination of AdaBoost classification with the colour analysis allowed a 96% detection rate, although 10% were false negatives and 3.5% of the fruits were not detected. Liu et al. [9] proposed an algorithm for the detection of greenhouse ripe tomatoes, where the Histogram of Oriented Gradients (HOG) descriptor was used to train a Support Vector Machine (SVM) classifier. A coarse-to-fine scanning method was developed to detect the fruit, followed by the proposed False Colour Removal (FCR) method to eliminate false-positive detections. The Non-Maximum Suppression (NMS) method was finally used in order to merge the overlapping results. The algorithm was able to detect the fruits with an accuracy of 94.41%. Wu et al. [26] developed a greenhouse ripe tomato detection algorithm for a harvesting robot, through a method that combines the analysis and selection of multiple features, a Relevance Vector Machine (RVM) classifier and a bi-layer classification strategy. The algorithm demonstrated an accuracy of 94.90%. Lili et al. [27], developing a greenhouse harvest robot for tomatoes, used the Otsu segmentation algorithm [40] for the automatic detection of ripe tomatoes, obtaining success rates of 99.3%.

In the most recent SoA, the interest in DL strategies has been growing [28,29,30,31,32,33]. This interest is due to the higher computability rate of the most recent computers and new edge computing devices dedicated to running DL models, as the TPU. Among the different DL architectures, the You Only Look Once (YOLO) models [41,42] seem to be the most common ones [28,29]. However, Convolutional Neural Network (CNN) structures also have their place due to their high accuracy, besides the long inference time [11]. This issue allows the appearance and use of the SSD adaptation of these models [11]. Among these adaptations, we can find the insertion of new layers (to increase the network resolution) and pruning of the output layers to fit the network classes, change the detection or feature extraction layers.

Xu et al. [28] improved the YOLOv3-tiny method to obtain a faster and more accurate detection of ripe tomatoes. The accuracy of the model was increased by improving the backbone network, and the image enhancement allowed better detection in more complex scenarios. The results obtained showed that the F1-score of the proposed model was 91.92%, which was 12% higher than the unmodified YOLOv3-tiny method. Liu et al. [29] used the YOLOv3 detection model to create the YOLOTomato model, which was possible to achieve due to the incorporation of the dense architecture for feature extraction and the replacement of the traditional R-box by the proposed C-box. In scenarios with moderate occlusions, the model obtained a detection rate of 94.58%, 4% more than in scenarios with severe occlusions. In order to overcome overlaps and occlusions, Sun et al. [30] developed a detection method based on CNN, more specifically the feature pyramid network method. By comparing this method with traditional Faster Region-based Convolutional Neural Network (R-CNN) models, the proposed method improved the detection rate from 90.7% to 99.5%. Mu et al. [31] built a tomato detection model capable of detecting green tomatoes in greenhouses, regardless of possible occlusions. The model uses a pre-trained Faster R-CNN structure with the deep convolutional neural network ResNet-101 based on the Common Objects in Context (COCO) dataset, which was then fine-tuned for tomato detection, reaching an accuracy of 87.83%.

As will be studied in this paper, the Deep Learning Single-Shot Multibox Detector (SSD) model promises a substantial improvement in fruit detection. Therefore, it has been increasingly studied, since it can capture the information of an object and its anti-interference, as well as directly complete the localisation and the classification task in just one step.

This improvement was demonstrated by de Luna et al. [32] who designed a computer visualisation system to evaluate the growth of tomato plants through the detection of fruits and flowers. Two deep learning models were used: R-CNN and SSD. The fruit detection accuracy of the R-CNN model was only 19.48%, while the SSD model showed a much higher detection rate of 95.99%. To detect cherry tomatoes in greenhouses, whether ripe, green or intermediate, Yuan et al. [33] developed an SSD-based algorithm. After building the datasets, they were used to train and develop network models. For studying the effect of the base network, one of the experiments was tested on different networks, such as VGG16, MobileNet and Inception V2. The results indicated that the Inception V2 network obtained the best performance with an accuracy of 98.85%.

### 2.2. Background

#### SSD Architecture

The Single-Shot MultiBox Detector (SSD) belongs to the One-Step Framework, also known as the Regression or Classification Based Framework, just like YOLO or RetinaNet [43,44]. With such frameworks, there is an explicit mapping between pixel values, bounding box coordinates and class probabilities, unlike Region Proposal-Based Frameworks, e.g., Faster RCNN. Therefore, compared to Faster RCNN and the same category of architectures, the SSD has lower inference times to the point of achieving real-time performance.

**Figure 2:**
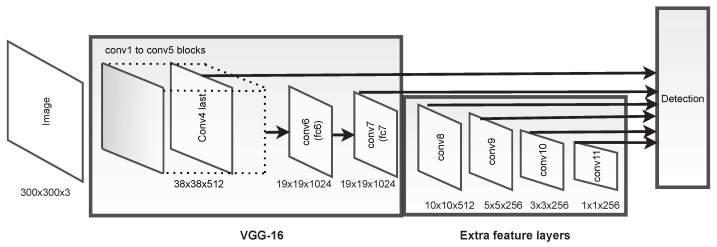
Scheme for the SSD architecture using VGG16 as the backbone. Adapted from ref. [11].

The SSD architecture, depicted in Figure 2, is composed of two main parts: feature extraction and object detection. For the first one, a state-of-the-art classification model is usually used (e.g., the VGG16 [45] network as in Figure 2), but others like ResNet [46] or MobileNet [47] are also possible. The feature extractor is called the backbone, and its purpose is to generate high-level feature maps from the input image. Besides the backbone, the SSD adds six extra feature maps with a decreasing spatial dimension; see Figure 2 [11,15].

**Figure 3:**
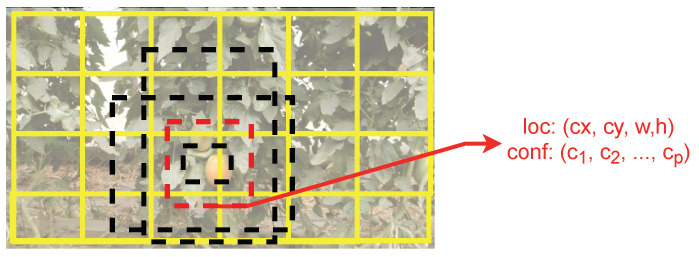
Anchor box shapes used in the SSD architecture. Adapted with permission from ref. [11].

For the second part, the SSD relies on a set of default anchors (i.e., bounding boxes) (Figure 3), with different aspect ratios and scales, thus reducing the possible amount of shapes that bounding boxes can assume [43]. A convolution layer is responsible for predicting, for each convolution operation and for each default anchor box, the location offset for that anchor and confidence scores for each class on the dataset. This convolution layer is applied to the extra feature maps. In the case of the VGG16 backbone, this layer is also applied to the Conv4_3 output [11]. Fusing the predictions made from the feature maps, each with a different resolution, allows detecting objects of different sizes. From Figure 2, using the feature maps towards the right will result in detecting larger objects, and vice versa [43].

In the end, many detections will be predicted, so Non-Maximum Suppression (NMS) is applied to keep the highest rated bounding boxes. As concerns training, a weighted sum between localization loss (e.g., smooth L1) and confidence loss (e.g., softmax) is used [43].

In order to improve SSD performance, the following measures can be used: choose default anchors with scales and aspect ratios according to the problem under analysis; perform data augmentation; and use hard negative mining. Even though, the SSD architecture performs worst in small object detection as these do not appear in all feature maps. This problem can be mitigated by using better feature extractor backbones (e.g., ResNet) and higher resolution input images [43].

## 3. Materials and Methods

**Figure 4:**
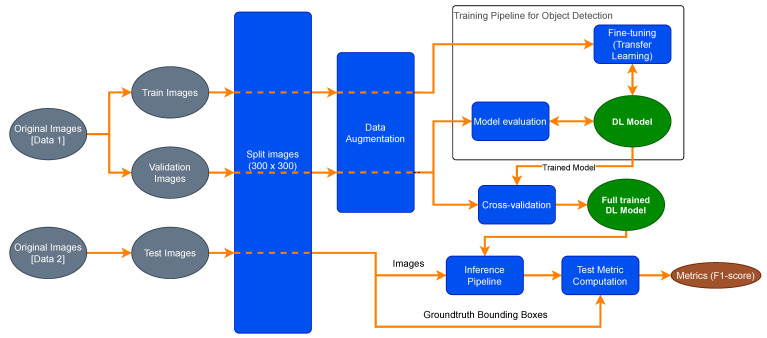
Overview of the performed methods. Training and evaluation pipeline.

Figure 4 reports an overview of the training and evaluation pipeline to reach a trained DL model. This architecture will be detailed in the following subsections.

### 3.1. Data Acquisition

Effective harvesting robots need to detect the harvesting target efficiently. In the current study, the robot intends to harvest tomatoes in a greenhouse tomatoes’ culture. Commonly used datasets, such as the COCO dataset [13], Open Images Dataset (OID) [14] and KITTI dataset [48], provide a large amount of data, but only OID has tomatoes in its data. However, these data are not representative of the kind of data class we intended to detect.

**Figure 5:**
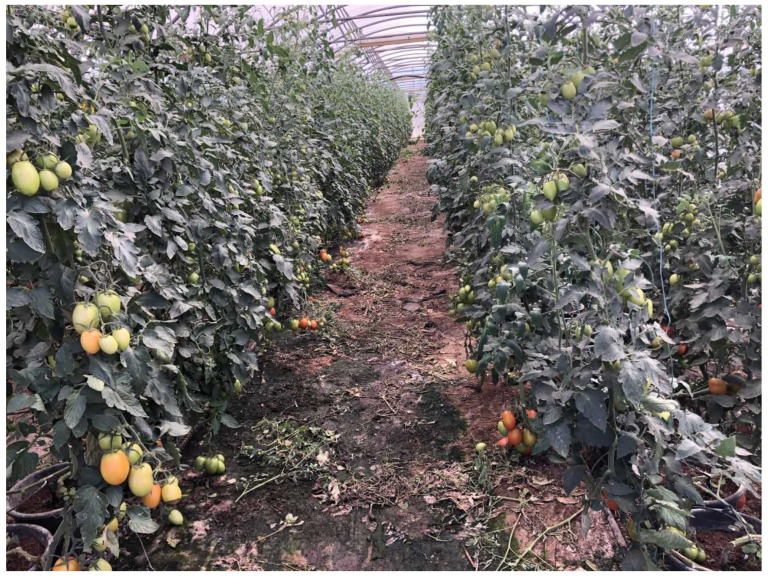
Greenhouses’ entrance.

To overcome these issues, a new image dataset of tomatoes was collected from a greenhouse at Barroselas in Viana do Castelo, Portugal. Although all the greenhouses on the campus are not equal, they have a similar configuration: six hedges of tomato plants with 0.9 m between-row spacing and 1.10 m of height (Figure 5), where the robot can travel. Tomatoes detached from the plant that have fallen to the ground are too ripe and should not be harvested.

**Figure 6:**
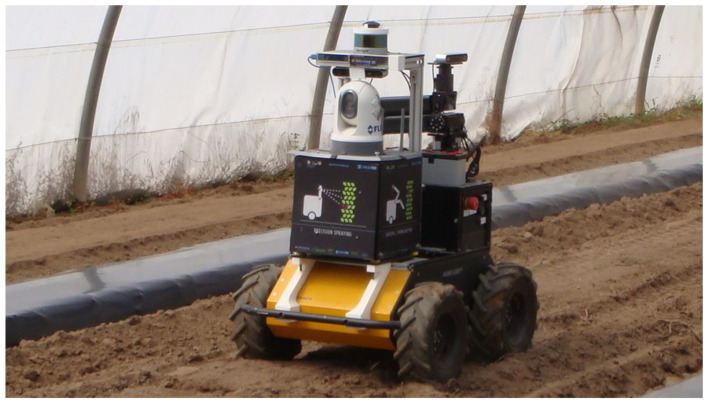
AgRob v16 inside an uncultivated greenhouse.

The mobile robot AgRob v16 (Figure 6) was used for recording images inside the greenhouses to increase the representativeness of the data. This robot is equipped with a set of sensors commonly used in robotic operations. Therefore, we obtained data acquired in the same conditions as a robot in a regular harvesting operation. At this stage, a human operator controlled, slowly, the robot through the different halls of the greenhouses, while the robot recorded, into a single ROSBag file, the information provided by its different sensors (cameras, IMU, LiDAR, among others). For the purposes of this study, only RGB images were relevant. The robot was moved along the crop row, keeping a distance between the robot and tomato plants from 0.4 m to 0.6 m.

As shown in Figure 6, the robot had two stereo cameras. The front camera was mainly used to localise the robot along the hall. However, for harvesting tomatoes, we intended to use a hand-eye strategy [49], which allowed a continuous refinement of the position of the robotic arm with respect to the tomatoes, using active perception [50] or gaze control mechanisms [51]. Therefore, the robot used the second stereo camera (ZED; see https://www.stereolabs.com/zed/, accessed on 25 November 2020) mounted on an anthropomorphic manipulator at the backside of the robot. The manipulator remained in the rest position as in Figure 6, i.e., looking sideward, towards the tomato plants, during the whole acquisition process. A Jetson Nano (see https://developer.nvidia.com/embedded/jetson-nano-developer-kit, accessed on 25 November 2020) Graphics Processing Unit (GPU) connected to the ZED camera in the robotic arm managed the camera and the collected images. After, the GPU sent the images to the onboard computer of the robot, to merge them with the remaining data, collected by the robot. In summary, a mobile robot collected images of the wall of tomato plants and recorded them as a video in an ROSBag file.

### 3.2. Dataset Generation

Most DL models are known as supervised ML algorithms. This specificity implies that the training mechanisms for DL models need to be fed with an annotated dataset. In the case of object detection, each annotation identifies its class, size and position. Some annotation types, like the Pascal VOC format, also include some additional features of the annotations as whether the target object is difficult to detect, occluded or truncated. For the current dataset, we used the Pascal VOC format, as used in the Pascal VOC Challenge [52], due to its simplicity, resuming the annotations for each image in a single XML file. In this case, we ignored the additional features because the TensorFlow1 Object Detection Pipeline ignores these features.

First of all, we converted the continuous video with the images of tomatoes into individual sequential images. To avoid high repetitiveness between images in the dataset, we acquired a frame every three seconds, assuring an overlapping ratio of around 60%. This process resulted in a dataset of 297 images with a resolution of 1280×720 px each.

All the images were manually labelled using the annotation tools CVAT [53] and LabelImg [54]. These tools allow better management of images and annotations, as well as a collaborative annotation. For this dataset, we only considered the class “tomato”, ignoring their ripeness, because of the low amount of ripened tomatoes (Figure 1), i.e., most of the tomatoes in the dataset were reddish or green (Figure 1).

**Figure 7:**
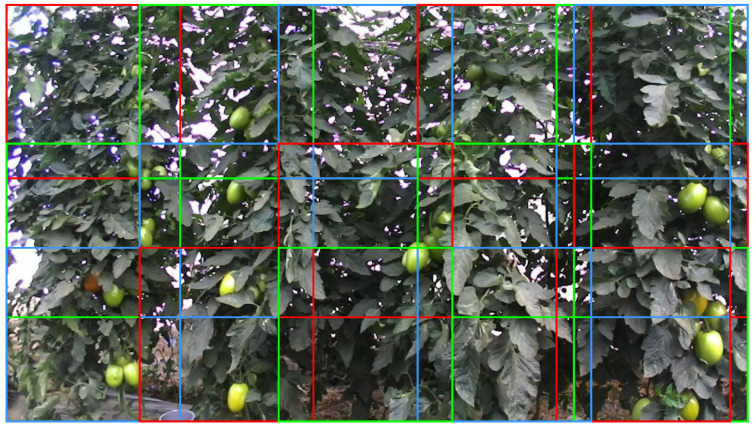
Images split into 300×300 px images with an overlapping ratio of 20%. The different colours are only for reference and distinguishing the different images.

We intended to use TPUs to detect tomatoes online inside the tomatoes’ greenhouses. However, TPUs are not compatible with all the ANN models. At the current time, they are only compatible with SSD models [11] and the tiny versions of You Only Look Once (YOLO) [42] model. Additionally, these models cannot process full-sized images, rescaling all the images before processing them. For instance, the pre-trained MobileNet v2 [15] can only process images of 300×300 px. Due to this, we split the original images into 300×300 px images, following the scheme represented in Figure 7, using pascal_voc_tools (see https://github.com/wang-tf/pascal_voc_tools, accessed on 8 September 2021). This splitting scheme considered the construction of sub-images of 300×300 px with a minimum overlapping ratio of 20% between sequential images. Splitting full-sized images of 1280×720 px to 300×300 px increased the dataset and the quality of the considered images for training. Therefore, this procedure increased the accuracy of the developed system. The Dataset now had 5365 images.

Some researches concluded that using augmentation strategies in the original images allowed increasing the dataset size and variability, contributing new information to it [55]. Different kinds of transformations may be applied to an image:

(i) rotation;
(ii) translation;
(iii) scaling;
(iv) hue modification;
(v) saturation;
(vi) blur;
(vii) noise;
(viii) others, even combinations of transformations.

**Figure 8:**
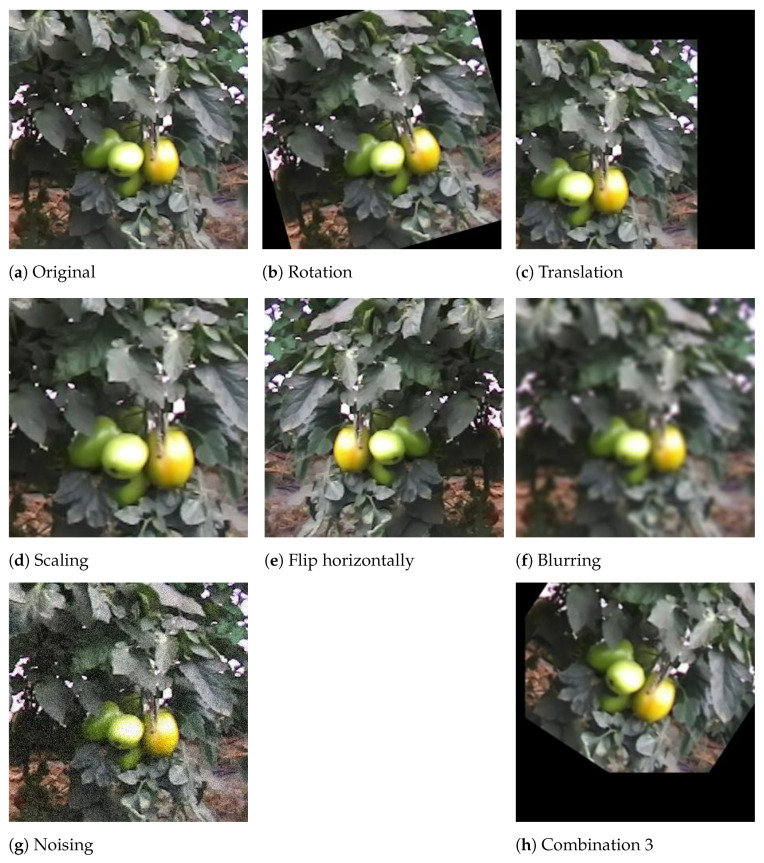
Example of augmentation applied to an image. (**h**) is the random combination of 3 of the other transformations.

Table 2 resumes the different applied transformations to images and their specifications. All transformations were applied with a random factor, to increase the variability of the data. Figure 8 exemplifies the use of augmentation in images. The augmented dataset resulted in a total of 23,021 images with 61,204 annotations of tomatoes.

For training purposes, we divided the dataset into two sets: training set and validation set. The training set contained 18,417 images with 49,100 annotations. The validation set had 4604 images with 12,104 annotations. For the evaluation and testing purposes of the trained models, an external set of annotated images was used. This set was acquired in the same conditions of the training and validation data, but in a different row of the tomatoes’ greenhouse. The original set of full-sized images with 1280×720 px had 250 images. For these data, the augmentation was not desired, but we still considered splitting the original images into smaller ones, with 300×300 px. This resulted in a set of 2737 images. The dataset then had 25,758 images. The acquired data are made publicly available at INESC TEC Research Data Repository (see https://rdm.inesctec.pt/ and http://www.doi.org/10.25747/pc1e-nk92, updated on 14 May 2021) [56].

### 3.3. Training and Evaluating SSD Models

The state-of-the-art has many frameworks for training and using deep learning algorithms [57,58,59,60,61]. However, TensorFlow [59], Darknet [60] and PyTorch [61] are the most known and used frameworks. Once we determined that our robot would use a TPU to detect tomatoes in the greenhouse, it needed a trained TensorFlow model.

TensorFlow [59] is a machine learning system that operates at a large scale and in heterogeneous environments. It is easily scalable and allows researchers and engineers to experiment with and test new ML algorithms. Therefore, TensorFlow supports a large variety of ML algorithms, focusing mainly on DL. TensorFlow is distributed as an open-source framework belonging to Google. It can run in a large variety of applications and devices as a centralised or distributed system.

During the development of the current evaluation of different models, only TensorFlow 1 had fully compatible tools to train and compile the models to the TPU. Therefore, the training and inference scripts used TensorFlow r1.15.0. Both scripts run in Google Colaboratory (Colab) notebooks (see http://colab.research.google.com) that offer free powerful GPU’s and TPU’s to train and infer deep learning models. The available GPUs varied each time we initialised a Colab session, but in this case, the NVIDIA Tesla T4 with a VRAM of 12 GB and 7.5 computation capability was the attributed GPU for all sessions.

For benchmarking purposes, we considered four pre-trained SSD models from the TensorFlow database (see https://github.com/tensorflow/models/blob/master/research/object_detection/g3doc/tf1_detection_zoo.md): SSD MobileNet v2, SSD Inception v2, SSD ResNet 50, and SSD ResNet 101 (see Appendix A, Table A1, for the location of the different models); and the YOLOv4 Tiny model. The first three models and YOLOv4 Tiny were previously pre-trained using the COCO dataset, and SSD ResNet 101 was pre-trained using OID. For fine-tuning the pre-trained models, we considered the default values of the pre-training pipeline, adjusting the batch size for the capacity of the available GPU, according to Table 3. All training sessions ran for 50,000 epochs, and an evaluation session occurred at every 50 epochs. The experiments with the different models proved that they did not need more than 50,000 epoch to converge to the best solution in the solution space. In some cases, the models converged after 30,000 epochs. The evaluation session at every 50 epochs followed the standard value used by the pre-trained models. These evaluation sessions allowed monitoring the evolution of the training. If the evaluation loss started to increase while the training loss decreased or remained constant, the deep learning model was over-fit to the training data. This situation did not happen to any trained networks.

YOLOv4 Tiny is not available for the TensorFlow framework, but is for the Darknet framework. The YOLOv4 Tiny model learned faster and only needed 2500 epochs for the training session. Darknet had no available validation sessions, so it was not considered.

The literature refers to many different evaluation metrics [52,62]. During the training process, we used the default pipeline metrics. However, as the COCO dataset and OID use different measures to evaluate the performance of the models, an additional common evaluation metrics pipeline was needed. For better benchmark SSD models, we preferred the metrics used by the Pascal VOC challenge [52] (precision × recall curve and mean average precision), as implemented by Padilla et al. [62], and complemented this evaluation with additional metrics:

(i) total recall;
(ii) total precision;
(iii) F1-score.

Recall (1) is the ability of the model to detect all the relevant objects, i.e., the ability of the model to detect all the detected bounding boxes of the validation set. Precision (2) is the ability of the model to identify only the relevant objects. F1-score (3) is the first harmonic mean between recall and precision. True Positives (TPs) are the correct detections of the ground truths. False Positives (FPs) are the objects that were improperly detected. False Negatives (FNs) are the undetected objects. The number of ground truths can be computed by the sum of the TPs and FNs (TP + FN), and the number of detections is the sum of the TPs and FPs (TP + FP). The detections are normally validated using the Intersection Over Union (IOU) metric [62] considering only the detections with an IOU ≥t. For the current benchmark, we considered t=50%.

(1) $$\mathrm{Recall}=\frac{\mathrm{TP}}{\mathrm{TP}+\mathrm{FN}}$$

(2) $$\mathrm{Precision}=\frac{\mathrm{TP}}{\mathrm{TP}+\mathrm{FP}}$$

(3) $$F_{1}=2\;\cdot \;\frac{\mathrm{Precision}\times \mathrm{Recall}}{\mathrm{Precision}+\mathrm{Recall}}$$

At the end of the training pipeline, all the models still had a free parameter: the confidence rate. The confidence rate is a value that features the certainty in the performed prediction. A prediction with a confidence rate of 50% determines that the network is 50% sure of the detected or classified object. Robust ANN tends to detect better and with higher confidences, but lower confidences can still have true positives. Therefore, optimising the confidence score is essential to optimise the network performance. The cross-validation technique is a useful technique to optimise this value. In the validation set, we removed all the augmentations and computed the F1-score for all the confidence thresholds from 0% to 100%, into steps of 1%. A confidence threshold considers all the confidence rates bigger than or equal to the stated one. The confidence threshold that optimises the F1-score is selected for the model’s normal operation.

We evaluated the four trained models to identify tomatoes using the test set. The whole inference process occurred on the Google Colab server, using a Tesla T4 GPU with a computation capability of 7.5.

## 4. Results and Discussion

This section evaluates the four SSD models and the YOLO model to detect tomatoes in greenhouses. As mentioned in Section 3.3, the trained models were evaluated using the metrics defined for the Pascal VOC challenge. Besides, some additional metrics were considered. In summary, we considered the following evaluation metrics:

- Recall × precision curve;
- mAP (mean Average Precision);
- Total recall;
- Total precision;
- F1-score;
- Inference time.

**Figure 9:**
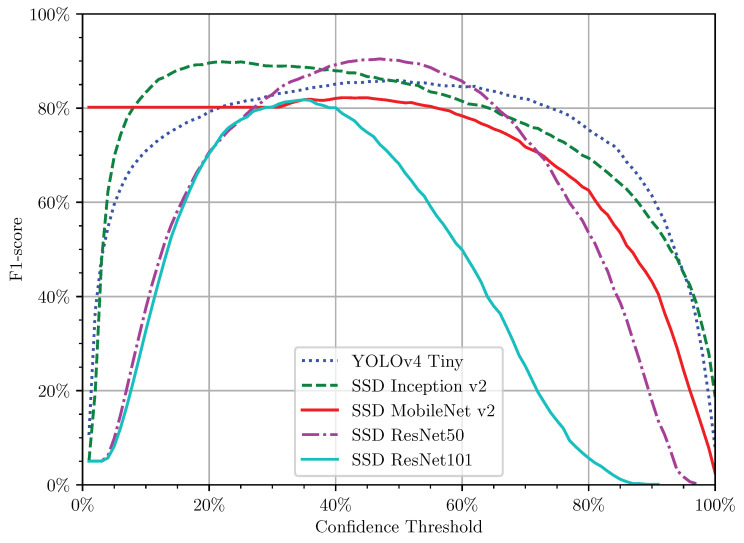
Evolution of the F1-score with the variation of the confidence threshold for all DL models in the validation set without augmentation.

**Figure 10:**
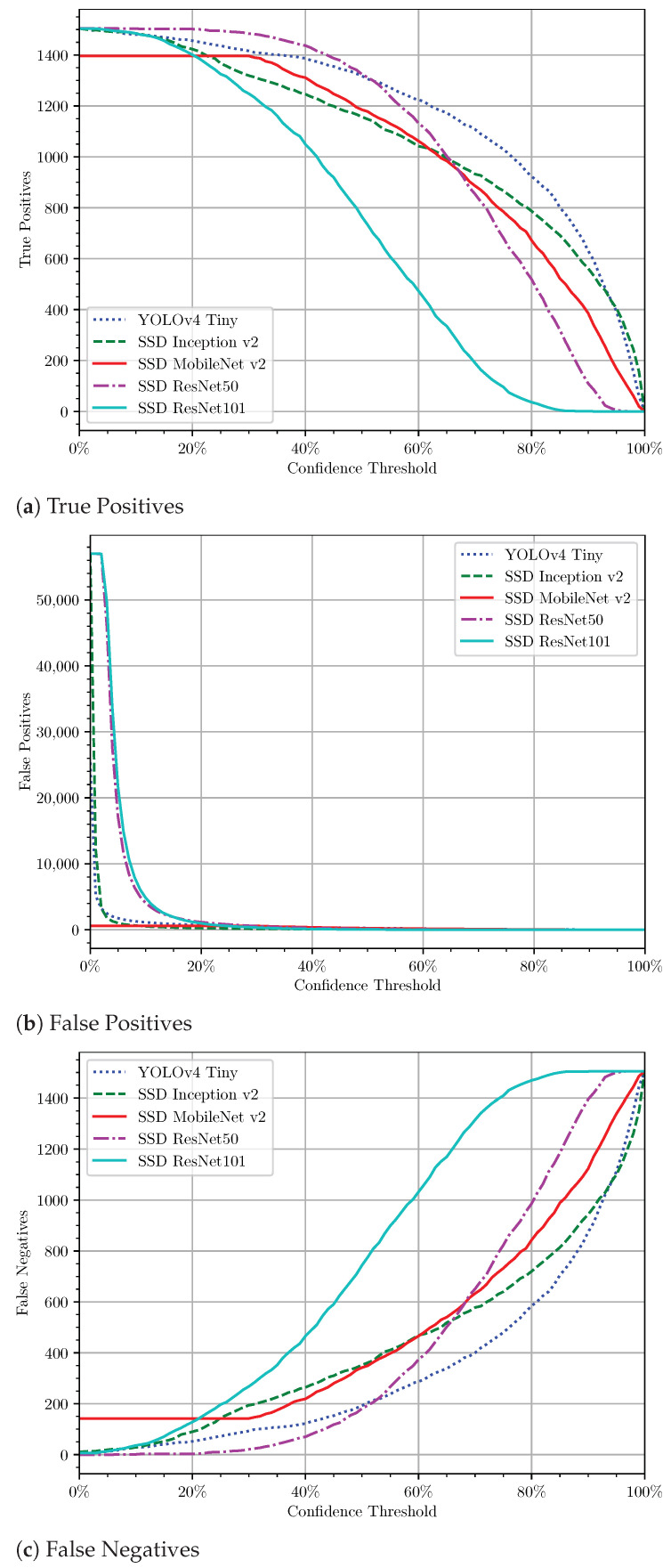
Evolution of the number of TPs, FPs, and FNs with the increase of the confidence threshold.

As mentioned in Section 3.3, before proceeding to evaluate the ANN’s performance, the models required defining the best confidence threshold first. This value is the confidence threshold that maximises the F1-score (Figure 9) because it find the best balance between the precision and recall, optimising the number of TPs while avoiding the FPs and FNs (Figure 10). Figure 9 reports the evolution of the F1-score with the variation of the confidence threshold for cross-validation. From this figure, we can quickly infer that some models have better behaviour than others. Models with flattened curves indicate higher confidence in their predictions and a low amount of FPs and FNs. Here, we can infer the maximum F1-score for each model and its confidence threshold (Table 4). These values are used to characterise the models for prediction purposes fully.

Particular attention should be given to SSD MobileNet v2. This ANN model almost has no FPs (Figure 10). This particularity is essential to avoid trying to harvest non-fruits and consequently damage the tree or the robot.

While the previous analysis provided the benchmark in the validation set, the following performed the test set’s performance analysis. This set was an independent collection of images and allowed understanding the generalisation capacity of the trained DL models. We started with a two-approach study and then converged to only the use of fully characterised models. First, we highlight the advantage of limiting the confidence rate.

**Figure 11:**
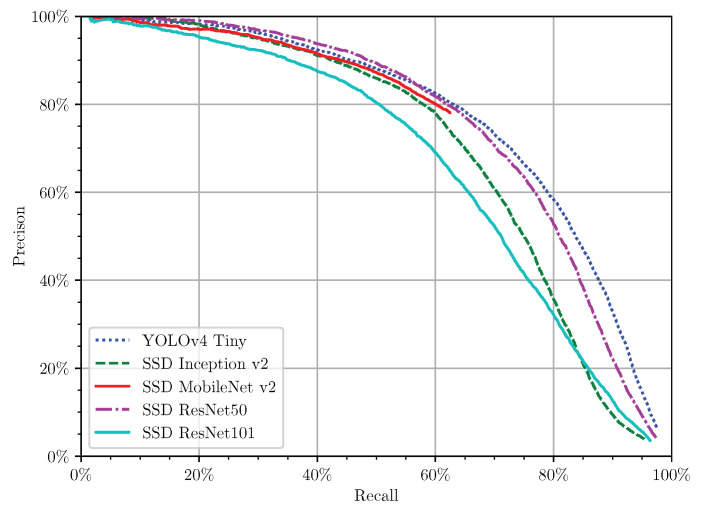
Precision × recall curve in the test set considering all the predictions.

Using all the predictions for detecting tomatoes in images of a greenhouse’s culture, a smooth precision × recall curve was built (Figure 11). This curve established the compromise between the recall rate and the precision rate, with the evolution of the prediction confidence score [62]. Higher confidence rates tend to have higher precision in their predictions, but a lower recall rate. All the models, except SSD MobileNet v2, had near a 100% recall rate, but in this stage, the precision was near 0%. The best performing model was the one with the highest Area Under the Curve (AUC) [62]. Therefore, from Figure 11, YOLOv4 Tiny and SSD ResNet 50 had similar results and were the best performing models. However, the low precision at higher recall rates and the lower total recall and F1-score (Table 5) mean that the models have much prediction noise and many false positives (knowing that SSD ResNet50 has the worst results). Therefore, while considering all the model predictions, using the F1-score as a balanced metric between the recall and the precision, SSD MobileNet v2 was the best performing model.

**Figure 12:**
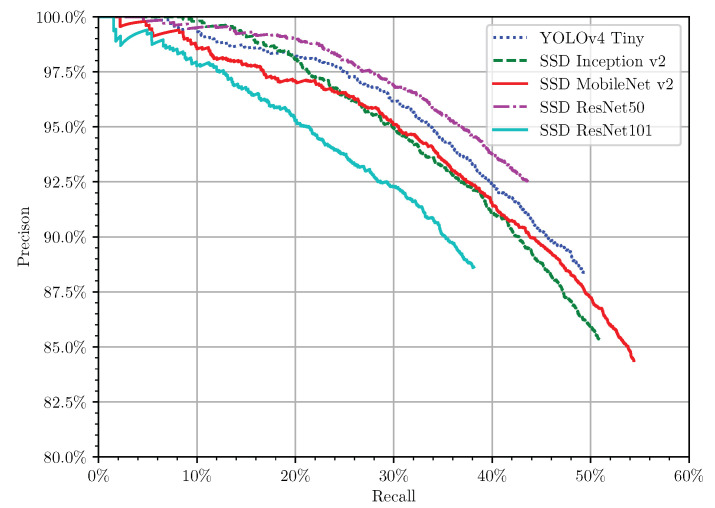
Precision × recall curve in the test set using the calibrated confidence threshold.

Most of the time, the low precision rates were due to the low confidence of predictions, as proven in the confidence threshold tuning process. If we performed an additional filtering process on the predictions, considering the best computed confidence threshold to maximise the F1-score in the validation set (Table 4 and Figure 9), the precision increased (Table 5). Doing this, also the precision × recall curve (Figure 12) was transformed through a truncation process. All the predictions had a precision rate higher than 80%, but a recall rate lower than 60%, as illustrated in Figure 12. For the fully characterised models, SSD MobileNet v2 continued to be the best performing model. However, despite the slightly lower results for real-time purposes, YOLOv4 Tiny seemed to have an interesting inference time. A particular note should be realised for this model. It is a quantised model (int8), while the others are not (float32). Therefore, a better analysis should be done to compare the results of all the models quantised. Finally, it was easier to conclude that SSD ResNet101 was a complex model for this problem and overfit, performing worst.

Quantisation is the process of mapping the continuous or large sets (here in the scope of 32 bit float) to a restricted set. In deep learning, it is common to map this number from the weights and bias of the neurons into 8 bit integers. The way edge devices compute makes this kind of ANN more suitable to run in real time. Typically, the performance of the ANN is irrelevantly penalised, but it becomes faster [63].

In general, all the illustrated models were not generic enough to successfully characterise the class tomato to detect all the tomatoes. The results became much worse between the validation set and the test set. Therefore, the amount and the variability of data should be increased.

**Figure 13:**
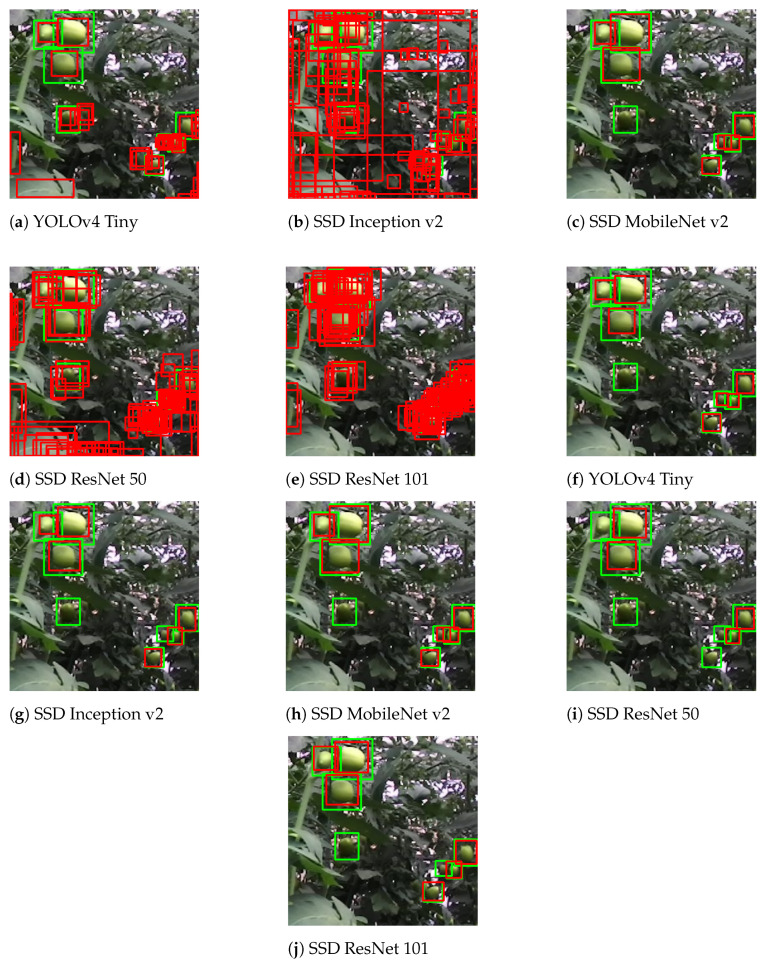
Comparison between using unfiltered images (**a**–**e**) and filtered images through the computed confidence threshold (**f**–**j**).

From Figure 13, it is easy to verify that using all the predictions from the inference process resulted in many false positives. Using filtered results by a threshold or similar process was significant for all models, except SSD MobileNet v2. This model, as demonstrated in Figure 9 and Figure 11, was well balanced between precision and recall and had a high confidence rate in its predictions, never reaching the situation of near 0% precision, i.e., any prediction was wrong. Additionally, this model also ensured a precision rate higher than 80%. Therefore, SSD MobileNet v2 can be used without any filtering process without compromising the results.

For better understanding the capability of the different models, we performed additional analysis of the results, considering representative images from the dataset for specific situations:

(i) darkened tomatoes;
(ii) occluded tomatoes;
(iii) overlapped tomatoes.

**Figure 14:**
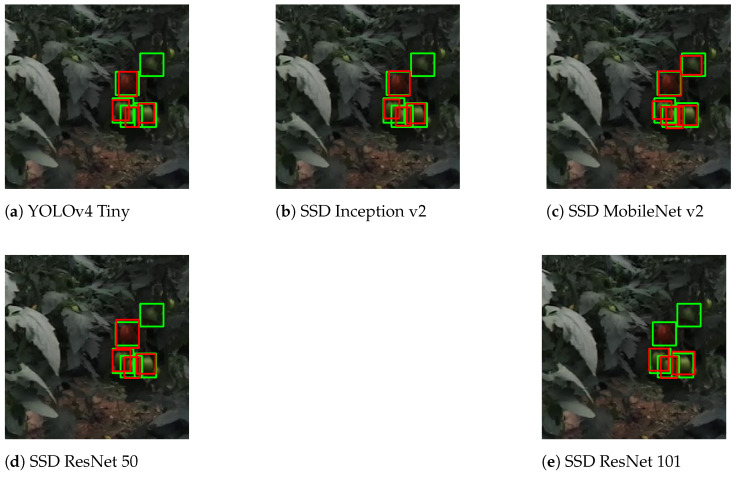
Result comparison for darkened images.

Figure 14 is a representative result of darkening tomatoes, which happens while the robot enters the greenhouse or when sun-protected in the shadow of other plants or leaves. For the current situation, all models had similar results. However, SSD MobileNet v2 performed slightly better, detecting one more tomato.

**Figure 15:**
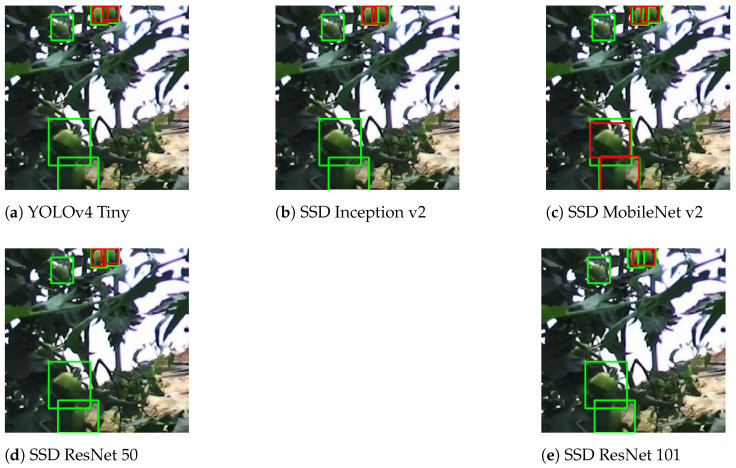
Result comparison for occluded tomatoes.

Occlusion refers to situations where a tomato is not fully visible. In these cases, tomatoes can be occluded by branches, stems, leaves or other tomatoes. Overlapping or clustering is a particular situation where other tomatoes occlude a tomato, and the detection system should detect both tomatoes. Figure 15 demonstrates a typical situation of occlusion by leaves. For this situation, SSD MobileNet v2 had the best generalisation of the network, detecting tomatoes that have less than 50% of their area occluded. All other networks did not detect the occluded tomatoes.

**Figure 16:**
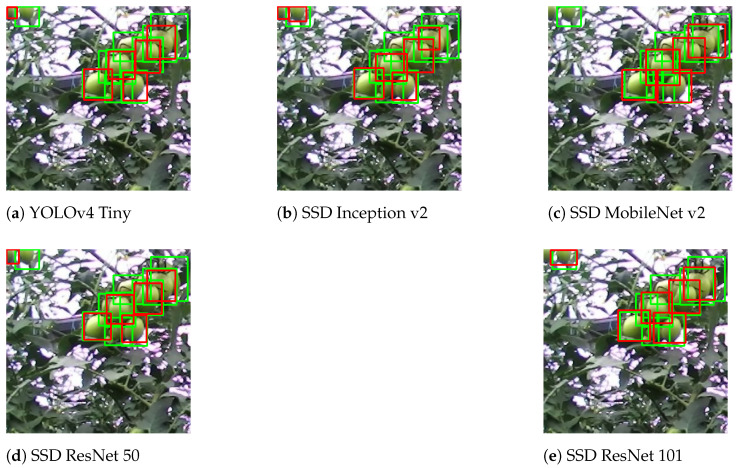
Result comparison for overlapped tomatoes.

Considering the case of clusters of tomatoes or overlapped tomatoes (Figure 16), all the DL models performed similarly. Therefore, all of them could be used equally for this situation.

In summary, SSD MobileNet v2 was the best performing model. It could handle all the situations, avoiding false positives. Besides, SSD MobileNet v2 was also the fastest network among the SSD models, inferring in 16 ms with a high-performance GPU. However, we cannot ignore the performance of YOLOv4. YOLOv4 Tiny was faster than the others because it is an quantised model, which significantly reduced the processing time.

In this work, we presented a real challenge, detecting tomato in the early ripening stage, where the colour feature was not so relevant, as stated in the literature review. We made this dataset public to support other works, and we analysed the most promising ANN models for edge computing. In these ANN models, most of the time, the confidence threshold was ignored or not tuned using clear criteria. We analysed this parameter, and we found there are ANN models where the performance can be significantly improved by tuning this parameter. With this work, we are able to move to the next stage, that is the deployment of these models into real robots and perception systems and benchmark against human labour—in terms of detection time, reliability and accuracy.

## 5. Conclusions

The choice of SSD architectures under other DL models is based on whether they can infer images quickly in TPUs. YOLO models are also the SoA, and researchers have made their tiny models compatible with TPUs. We benchmarked four pre-trained SSD models from the TensorFlow database for detecting tomatoes and YOLOv4 Tiny from the Darknet database. The dataset was acquired inside the tomatoes’ greenhouse using a stereo camera. The dataset was made publicly available at INESC TEC Research Data Repository (see https://rdm.inesctec.pt/ and https://doi.org/10.25747/pc1e-nk92, updated on 14 May 2021) [56].

The results proved that SSD MobileNet v2 was the best generalised and best performing model. Besides, it had the lowest ratio of FPs and performed the computation quickly. Although, if the inferring time was not fast enough, YOLOv4 Tiny also had impressive results and could process an image in about 5 ms.

The worst performing DL model was SSD ResNet 101, with an F1-score of 38.13%, and inferring images in 60 ms. SSD ResNet 101 is a complex model, so it needed more time to infer images. This complexity of the model favoured overfitting when the data were not enough or not representative of the class. Therefore, future work for this particular model is needed:

(i) increasing the representativeness and the size of the dataset;
(ii) adding regularisation to the model, penalising complex models.

The additional future work will focus on:

1. creating new sub-classes to consider different tomatoes’ contexts, as occluded tomatoes or darkened tomatoes;
2. evaluating the performance of the detection system in on-time conditions inside the greenhouses;
3. adding the capability to distinguish and evaluate the ripeness of tomatoes for harvesting procedures.

## Figures and Tables

**Table 1:** Algorithms, methods and techniques proposed by different authors regarding tomato detection at different ripeness levels (N/A—Not Available).

Method	Tomato Ripeness	Accuracy	Inference Time	Authors/Year
L*a*b* colour space and K-means clustering	Ripe	N/A	10.10 s	Yin et al. [16] 2009
L*a*b colour space and bi-level partition fuzzy logic entropy	Ripe	N/A	N/A	Huang et al. [17] 2012
L*a*b colour space and Threshold algorithm	Green, intermediate and ripe	93%	N/A	Zhao et al. [25] 2016
RGB, HSI and YIQ colour spaces and morphological characteristics	Ripe	96.36%	N/A	Arefi et al. [18] 2011
RGB colour space images into an HSI colour model	Ripe	83.9%	4 s	Feng et al. [35] 2015
	Ripe	83.9%	4 s	Feng et al. [35] 2015
RGB colour space into an HSI colour space, threshold method and Canny operator	Ripe	N/A	N/A	Zhang [19] 2015
R component of the RGB images and Sobel operator	Ripe	Clustered tomatoes: 87.5%	N/A	Benavides et al. [20] 2020
		Beef tomatoes: 80.8%		
HSV colour space and watershed segmentation method	Ripe	81.6%	N/A	Malik et al. [21] 2018
Mathematical morphology and Fuzzy C-Means-based method	Ripe	N/A	N/A	Zhu et al. [22] 2012
Mathematical morphology, difference and iterative erosion course	Ripe	50 cm–87.5% 30 to	N/A	Xiang et al. [23] 2013
Normalised colour		70 cm–58.4%		
Pixel-based segmentation, blob-based segmentation and X-means clustering	Green, intermediate and ripe	88%	N/A	Yamamoto et al. [24] 2014
Haar-like features of grey-scale image and AdaBoost classifier	Ripe	96%	N/A	Zhao et al. [39] 2016
Histograms of oriented gradients and SVM	Ripe	94.41%	N/A	Liu et al. [9] 2019
	Ripe	94.41%	N/A	Liu et al. [9] 2019
Analysis and selection of multiple features, RVM and bi-layer classification strategy	Ripe	94.90%	N/A	Wu et al. [26] 2019
Otsu segmentation algorithm	Ripe	99.3%	N/A	Lili et al. [27] 2017
Improved YOLOv3-tiny method	Ripe	F${}_{1}=$ 91.92%	N/A	Xu et al. [28] 2020
YOLOv3 detection model to create the proposed YOLOTomato model	Green, intermediate and Ripe	94.58%	N/A	Liu et al. [29] 2020
Feature pyramid network	Green, intermediate and Ripe	99.5%	N/A	Sun et al. [30] 2020
Faster R-CNN structure with the deep CNN ResNet-101	Green	87.83%	N/A	Mu et al. [31] 2020
Comparison: R-CNN vs. SSD	Green, intermediate and Ripe	R-CNN: 19.48%	N/A	de Luna et al. [32] 2020
		SSD: 95.99%		
SSD-based algorithm used to train and develop network models such as VGG16, MobileNet, Inception V2	Green, intermediate and Ripe	Best performance is Inception V2 ( 98.85%)	N/A	Yuan et al. [33] 2020

**Table 2:** Transformations applied to the images of the split dataset for data augmentation and the characteristics of those transformations.

Transformation	Value
Rotation	−60° to 60°
Scaling	50% to 150%
Translation	0% to 30% left or right
Flip	Image mirroring
Blur (Gaussian Filter)	N(0,1to3)
Gaussian Noise	N(0,0.03·255to0.07·255)
Combination3	Random combination of three of the previous transformations with random values

**Table 3:** Training batch size for each model.

SSD Model	Batch Size
SSD MobileNet v2	24
SSD Inception v2	32
SSD ResNet 50	8
SSD ResNet 101	8
YOLOv4 Tiny	64

**Table 4:** Confidence threshold for each DL model that optimises the F1-score metric.

	Confidence ⩾	F1-Score
YOLOv4 tiny	49%	85.92%
SSD Inception v2	21%	89.85%
SSD MobileNet v2	40%	82.22%
SSD ResNet 50	46%	90.46%
SSD ResNet 101	34%	81.75%

**Table 5:** Results of the different SSD and YOLO models over many metrics, considering all the predictions and the best computed confidence threshold.

Model	Confidence ⩾	Inference Time	mAP	Precision	Recall	F1
YOLOv4 Tiny	0%	4.87 m s	77.19%	6.38%	97.52%	11.98%
SSD Inception v2	0%	24.75 m s	70.39%	3.53%	95.82%	6.82%
SSD MobileNet v2	0%	16.44 m s	57.99%	78.07%	62.44%	69.39%
SSD ResNet50	0%	47.78 m s	75.74%	3.6%	97.62%	6.94%
SSD ResNet101	0%	59.78 m s	66.88%	3.55%	96.32%	6.85%
YOLOv4 Tiny	49%	4.87 m s	47.48%	88.39%	49.33%	63.32%
SSD Inception v2	21%	24.75 m s	48.54%	85.31%	50.93%	63.78%
SSD MobileNet v2	40%	16.44 m s	51.46%	84.37%	54.40%	66.15%
SSD ResNet50	46%	47.78 m s	42.62%	92.51%	43.59%	59.26%
SSD ResNet101	34%	59.78 m s	36.32%	88.63%	38.13%	53.32%

## Data Availability

The data presented in this study are openly available in INESC TEC Research Data Repository at DOI:10.25747/PC1E-NK92, reference number ii-2021-001.

## Abbreviations

The following abbreviations are used in this manuscript:

AI	Artificial Intelligence
ANN	Artificial Neural Network
COCO	Common Objects in Context
DP	Deep Learning
IMU	Inertial Moment Unit
GPU	Graphics Processing Unit
HSI	Hue, Saturation and Intensity
HSV	Hue, Saturation and Value
LiDAR	Light Detection and Ranging
mAP	mean Average Precision
ML	Machine Learning
RGB	Red, Green and Blue
RGB-D	Red, Green, Blue and Depth
ROS	Robotics Operating System
RVM	Relevance Vector Machine
SSD	Single-Shot MultiBox Detector
SVM	Support Vector Machine
TPU	TensorFlow Processing Unit
VRAM	Video Random-Access Memory
YOLO	You Only Look Once

## Appendix A

**Table A1:** Model location in TensorFlow and Darknet databases. All SSD models are in the TensorFlow Models database at http://download.tensorflow.org/models/object_detection/filename. YOLOv4 Tiny is in the Darknet database at https://github.com/AlexeyAB/darknet/releases/download/filename.

SSD Model	File Name
SSD MobileNet v2	ssd_mobilenet_v2_coco_2018_03_29.tar.gz
SSD Inception v2	ssd_inception_v2_coco_2018_01_28.tar.gz
SSD ResNet 50	ssd_resnet50_v1_fpn_shared_box_predictor_640x640_coco14_sync_2018_07_03.tar.gz
SSD ResNet 101	ssd_resnet101_v1_fpn_shared_box_predictor_oid_512x512_sync_2019_01_20.tar.gz
YOLOv4 Tiny	darknet_yolo_v4_pre/yolov4-tiny.conv.29
